# Comprehensive Genotoxicity and 28‐Day Oral Toxicity Evaluation Reveal Safety of a Standardized *Anisomeles indica*‐Containing Powder

**DOI:** 10.1155/bmri/9966654

**Published:** 2026-03-13

**Authors:** Yng-Tay Chen, Yu-Ru Chen, Fuu-Jen Tsai, Ter-Hsin Chen

**Affiliations:** ^1^ Graduate Institute of Food Safety, College of Agriculture and Natural Resources, National Chung Hsing University, Taichung City, Taiwan, nchu.edu.tw; ^2^ Department of Food Science and Biotechnology, College of Agriculture and Natural Resources, National Chung Hsing University, Taichung City, Taiwan, nchu.edu.tw; ^3^ Department of Post-Baccalaureate Medicine, College of Medicine, National Chung Hsing University, Taichung City, Taiwan, nchu.edu.tw; ^4^ Genetic Center, Department of Medical Research, China Medical University Hospital, Taichung, Taiwan, cmu.edu.cn; ^5^ School of Chinese Medicine, College of Chinese Medicine, China Medical University, Taichung, Taiwan, cmu.edu.cn; ^6^ Graduate Institute of Veterinary Pathobiology, National Chung Hsing University, Taichung, Taiwan, nchu.edu.tw; ^7^ Research Center for Animal Medicine, National Chung Hsing University, Taichung, Taiwan, nchu.edu.tw

**Keywords:** *Anisomeles indica*, genotoxicity, NOAEL, repeated dose 28-day oral toxicity

## Abstract

*Anisomeles indica* is widely used in traditional medicine and functional foods; however, its toxicological safety has not been systematically evaluated. This study evaluated the genotoxicity and subacute oral toxicity of a standardized *A. indica*‐containing powder (AIHP) using OECD‐compliant test guidelines. Genotoxicity was assessed via the bacterial reverse mutation test (OECD 471), in vitro chromosomal aberration test (OECD 473), and in vivo micronucleus assay in mice (OECD 474). A repeated‐dose 28‐day oral toxicity study (OECD 407) was performed in Sprague–Dawley rats at doses of 2000, 4000, and 8000 mg/kg/day. AIHP did not induce mutagenicity, chromosomal aberrations, or micronucleus formation in any assay. No treatment‐related mortality, clinical signs, or adverse changes in body weight, hematology, serum biochemistry, organ weights, urinalysis, or histopathology were observed. The no‐observed‐adverse‐effect level (NOAEL) was established at 8000 mg/kg/day. These results support the toxicological safety of AIHP and its suitability for use as a health food ingredient.

## 1. Introduction

*Anisomeles indica* (L.) Kuntze, a member of the Lamiaceae family, has long been revered in Asian traditional medicine for its impressive efficacy in treating gastrointestinal ailments such as gastric ulcers and inflammation associated with *Helicobacter pylori* (*H. pylori*) infection [1–5]. The potent bioactive compounds in *A. indica* extracts—including apigenin, ovatodiolide, *β*‐sitosterol, and acteoside have been scientifically shown to alleviate inflammation, inhibit tumor cell growth, and effectively suppress *H. pylori* infection [6–9]. These pharmacological actions extend to the treatment of gastrointestinal, liver, and inflammatory skin diseases, underscoring the therapeutic versatility of this herbal remedy. Notably, our previous research identified acteoside as a key active constituent, demonstrating its powerful protective effects against ethanol‐induced gastric ulcers by modulating the I*κ*B‐*α* and NF‐*κ*B pathways [5].

Excess gastric acid secretion is a critical factor in the development of serious gastrointestinal disorders, often exacerbated by infection with *H. pylori*. This resilient bacterium not only thrives in acidic environments but also intensifies inflammation in the stomach by secreting potent toxins and proinflammatory agents [10, 11]. Mounting evidence links *H. pylori* infection directly to the onset of gastric ulcers and chronic gastritis, highlighting the urgent need for effective interventions [12]. Furthermore, excessive gastric acid can rapidly compromise the stomach’s protective mucosal barrier—composed of mucus and bicarbonate ions—rendering it vulnerable to erosion and ulceration [13, 14]. Once this natural defense is weakened, the risk of ulcer formation escalates substantially [15]. Compounding the problem, heightened gastric acidity creates an environment prone to oxidative stress, leading to a surge of free radicals and other damaging oxidative molecules that further deteriorate cellular integrity and drive harmful inflammatory responses [16, 17]. Addressing these interconnected mechanisms is essential for restoring and protecting gastrointestinal health.

In this study, we conducted a comprehensive safety evaluation of *A. indica* HP813‐containing powder (AIHP) using rigorous genotoxicity assays and a 28‐day repeated‐dose oral toxicity study, aiming to provide strong evidence for its safety and potential as an innovative health food ingredient.

## 2. Material and Methods

### 2.1. Sample

AIHP is a standardized powder formulation supplied by SYI Biotechnology (Taichung, Taiwan), composed of *A. indica* extract powder blended with soluble corn fiber and xylo‐oligosaccharides as excipients to improve stability and dispersibility. The *A. indica* extract powder was prepared using a food‐grade extraction process. Acteoside was chosen as a phytochemical marker based on prior pharmacological studies and quantified at 55 *μ*g per 2000 mg of AIHP to ensure batch consistency. The product was manufactured under controlled conditions, and all test materials used in this study originated from the same production batch. Chemical characterization, including identification of acteoside as a marker compound, was based on previously published HPLC analyses of *A. indica* extracts and the supplier’s quality control documentation [4]. No independent chromatographic analysis was conducted in the present study.

### 2.2. Genotoxicity Test

#### 2.2.1. Bacterial Reverse Mutation Test (Ames Test)

This experimental procedure rigorously adhered to the OECD 471 test guideline [18], underscoring the scientific credibility of our approach. Five carefully selected bacterial strains were employed in the reverse mutation (Ames) test: *Salmonella typhimurium (S. typhimurium)* TA98, TA100, TA1535, TA1537, and *Escherichia coli* (*E. coli*) WP2 uvrA (pKM101). TA98, TA100, and WP2 uvrA (pKM101) were sourced from the highly reputable Bioresources Collection and Research Center (BCRC), Food Industry Research and Development Institute, Hsinchu, Taiwan. To ensure comprehensive safety profiling, AIHP underwent toxicity testing against all five bacterial strains using a robust range of dosages (1.25, 2.5, and 5 mg per plate). Bacterial cultures were meticulously prepared, serially diluted, and combined with the sample solution before being plated on nutrient agar and incubated for 24 h. Notably, results demonstrated that even the highest tested concentration (5 mg/plate) of AIHP exhibited no toxicity in any strain, supporting its safety. The Ames test was conducted both with and without metabolic activation using the S9 fraction, further enhancing the reliability of our findings. AIHP was examined at multiple concentrations (0.3125, 0.625, 1.25, 2.5, and 5 mg/plate), with the experimental setup thoughtfully designed to meet the highest scientific standards. A mixture of 2 mL of 0.75% soft agar, 100 *μ*L of bacterial suspension, 200 *μ*L of S9 mix, 100 *μ*L of sample or positive control, and 200 *μ*L of histidine/biotin solution was poured onto minimal glucose agar plates. After solidification, samples were incubated at 37°C for 48 h. A substance was classified as mutagenic only if the revertant colony count in the test group exceeded twice that of the negative control, ensuring rigorous and meaningful interpretation of results.

#### 2.2.2. Chromosomal Aberration Test

This experimental procedure rigorously followed the internationally recognized OECD 473 test guideline [19], ensuring the highest standards of scientific reliability. Chinese hamster ovary K1 (CHO‐K1) cells, sourced from the esteemed BCRC, were cultured under optimal conditions in Ham’s F‐12 medium with 10% FBS and 1% penicillin/streptomycin, maintained at 37°C with a 5% CO_2_ incubator to guarantee cell health and experimental consistency. Initial cytotoxicity screening and IC_50_ determination via the MTT assay provided a solid foundation for dose selection. Notably, a 24‐h treatment with 5 mg/mL of AIHP reduced CHO‐K1 cell viability to 51.25% ± 2.32%, validating this as the maximum test concentration for the chromosomal aberration assay. To ensure comprehensive assessment, AIHP was evaluated at a broad range of concentrations (0.3125, 0.625, 1.25, 2.5, and 5 mg/mL), both with and without metabolic activation (S9). CHO‐K1 cells (2 × 10^5^ cells/mL) were precisely seeded, and all treatments—including gold‐standard positive controls (mitomycin C and cyclophosphamide) were meticulously administered to maximize result validity. Treatment regimens were carefully designed as follows: 21 h without S9 or 3 h with S9 followed by 18‐h recovery. Colchicine (10 *μ*g/mL) was strategically added 3 h before harvest to ensure optimal metaphase analysis. Following hypotonic treatment with 0.6% KCl and fixation in methanol: acetic acid (3:1) at −20°C for at least 24 h, slides were expertly prepared and Diff‐Quik stained. For robust statistical power, 300 metaphase cells per treatment were analyzed for structural chromosomal aberrations. Structural aberrations, including chromatid breaks, chromosome breaks, exchanges, and fragments, were recorded. Chromosomal gaps were noted but were not included in the total aberration frequency, in accordance with standard cytogenetic scoring criteria. To support interpretation of aberration frequencies, cytotoxicity was assessed by evaluating mitotic activity, and concentrations inducing excessive cytotoxicity were excluded from analysis.

#### 2.2.3. ICR Mice Erythrocyte Micronucleus Test

This experimental procedure was meticulously designed in full accordance with the internationally acclaimed OECD 474 test guideline [20], and received official approval from the Institutional Animal Care and Use Committee of National Chung Hsing University (permit number: IACUC 113‐001), underscoring the study’s scientific integrity and ethical rigor. Twenty‐five healthy male ICR mice, aged 5 weeks, were carefully sourced from BioLASCO Taiwan (Yilan, Taiwan) and randomly assigned to five well‐defined groups (*n* = 5 per group): negative control, positive control (cyclophosphamide, 60 mg/kg), and low‐, medium‐, and high‐dose AIHP groups (2000, 4000, 8000 mg/kg, administered via oral gavage at 10 mL/kg). Blood samples were precisely collected at 48 and 72 h post‐treatment through the retro‐orbital sinus under isoflurane anesthesia, ensuring both animal welfare and data accuracy. Smears were expertly stained with 0.1% acridine orange to facilitate sensitive detection. For each animal, at least 2000 reticulocytes (RETs) were scored for the presence of micronuclei. The frequency of micronucleated reticulocytes (Mn‐RETs) was reported as the number of Mn‐RETs per 1000 RETs. Additionally, the proportion of RETs among total red blood cells (RETs/1,000 RBCs) was determined as an indicator of bone marrow toxicity or cytotoxicity, and using advanced fluorescence microscopy (520 nm barrier filter; 450–490 nm excitation), ensuring a highly sensitive and reliable assessment of genotoxic effects. This comprehensive and ethically robust design reinforces the credibility and persuasive power of the study’s findings.

#### 2.2.4. Repeated Dose 28‐Day Oral Toxicity Study in SD Rats

A total of 80 healthy, 5‐week‐old Sprague–Dawley rats of both sexes were meticulously acquired from BioLASCO Taiwan (Yilan, Taiwan) and randomly allocated into four scientifically defined groups: a control group and three AIHP treatment groups receiving low (2000 mg/kg), medium (4000 mg/kg), and high (8000 mg/kg) doses, respectively. Dose selection for the 28‐day oral toxicity study was based on OECD Test Guideline 407 and the intended use of AIHP as a food‐derived ingredient. High doses (2000–8000 mg/kg/day) were chosen to establish a conservative margin of safety. The highest dose reflects a worst‐case exposure scenario and enables identification of potential target organ toxicity. Based on body surface area conversion, the NOAEL of 8000 mg/kg/day in rats corresponds to a human equivalent dose of approximately 1300 mg/kg/day. This provides a substantial margin of exposure compared with anticipated human consumption levels. AIHP was precisely administered via oral gavage once daily for 28 consecutive days (10 mL/kg), ensuring controlled and consistent dosing. Throughout the study, animals were closely monitored for clinical signs, with body weights systematically recorded each week to track health and growth. On Day 28, a comprehensive 24‐h urine sample was collected and analyzed using an advanced automatic urinalysis analyzer (AE‐4020, Sysmex, Kobe, Japan), providing key insights into renal and metabolic health. On Day 29, under isoflurane anesthesia to maximize animal welfare, blood and major organs were carefully collected for in‐depth analyses. Hematological parameters were accurately measured using a state‐of‐the‐art analyzer (Sysmex XE2100), and serum biochemistry was evaluated with a high‐precision system (ADVIA 1800, Siemens, NY, United States). Major organs were expertly excised, weighed, and subjected to thorough gross morphological assessments. Subsequent fixation in 10% neutral buffered formalin and hematoxylin and eosin staining enabled detailed microscopic examination of tissue health. Every procedure was rigorously conducted in accordance with the OECD Test Guideline No. 407 (Repeated Dose 28‐Day Oral Toxicity in Rodents) [21], and received ethical clearance from the Institutional Animal Care and Use Committee of National Chung Hsing University (permit number: IACUC 113‐001), reinforcing the scientific credibility and ethical integrity of this comprehensive toxicity assessment.

#### 2.2.5. Statistical Analysis

All statistical analyses were performed using methods appropriate to the nature and distribution of the data. Continuous variables (e.g., body weight, food consumption, hematology, and serum biochemistry parameters) were first assessed for normality and homogeneity of variance. When these assumptions were met, data were analyzed using one‐way analysis of variance (ANOVA) followed by Tukey’s post hoc test for multiple comparisons. For comparisons between two groups, a Student’s *t*‐test was applied, as appropriate. Categorical or semi‐quantitative data, including urinalysis parameters expressed as incidence or percentages, do not meet the assumptions required for parametric statistical testing. Therefore, these data were evaluated descriptively using frequency‐based comparisons and were not subjected to ANOVA or *t*‐tests. This approach avoids inappropriate statistical inference and aligns with standard toxicological practice. All statistical tests were two‐sided, and differences were considered statistically significant at *p* < 0.05 for continuous variables. Statistical significance was interpreted in conjunction with biological relevance and toxicological context to ensure robust conclusions.

## 3. Results

### 3.1. AIHP Did Not Induce Reverse Mutations in *S. typhimurium* or *E. coli*

The bacterial toxicity assay provided robust evidence that *S. typhimurium* strains TA98, TA100, TA1535, TA1537, and *E. coli* WP2 uvrA (pKM101) experienced absolutely no cytotoxic response to AIHP, even at the highest tested concentration of 5 mg/plate. Building on this, a comprehensive Ames test was performed using all five strains, with meticulous evaluation at the maximum dose, both with and without metabolic activation by the S9 fraction. Remarkably, no increase in revertant colony counts was detected at any concentration of AIHP, regardless of S9 activation (Table 1), affirming the absence of mutagenic effects. In stark contrast, positive control groups exhibited a striking elevation in revertant colony counts, far surpassing negative controls and confirming the exceptional reliability and validity of the assay. These findings collectively underscore the outstanding genetic safety of AIHP. Results demonstrated that AIHP produced no statistically significant differences compared with controls. All values remained within historical reference ranges and showed no dose–response relationship.

**Table 1:** Revertant changes of AIHP in *S. typhimurium* TA98, TA100, TA1535, TA1537 and *E. coli* WP2 *uvr* A. (pKM101) mutagenicity test.

Group	Number of revertant (colony/plate)				
	*S. typhimurium*				*E. coli*
	TA98	TA100	TA1535	TA1537	WP2 *uvr* A (pKM101)
Without S9					
Control^a^	39.3 ± 2.9^c^	120.0 ± 8.6	12.0 ± 0.8	12.3 ± 5.6	166.3 ± 3.1
PC^b^	1424.6 ± 697.7^∗^	637.3 ± 66.2^∗^	474.7 ± 18.7^∗^	2660.6 ± 916.7^∗^	404.0 ± 2.8^∗^
AIHP (mg/plate)					
0.3125	40.0 ± 2.4	116.0 ± 10.2	13.3 ± 3.9	9.7 ± 2.5	167.3 ± 2.1
0.625	33.3 ± 4.3	121.7 ± 1.7	14.3 ± 2.4	9.3 ± 1.7	163.7 ± 8.1
1.25	37.7 ± 1.7	118.7 ± 3.3	10.3 ± 2.6	14.0 ± 2.9	164.0 ± 7.8
2.5	41.3 ± 1.2	130.7 ± 4.1	14.0 ± 0.8	11.7 ± 1.2	167.0 ± 8.3
5	41.7 ± 2.1	123.7 ± 3.1	13.6 ± 7.9	9.3 ± 0.5	170.7 ± 4.1
With S9					
Control	30.0 ± 5.9	127.7 ± 1.7	18.7 ± 1.7	10.0 ± 1.4	178.0 ± 4.5
PC^b^	1359.0 ± 444.6^∗^	1505.3 ± 55.3^∗^	125.3 ± 6.8^∗^	130.0 ± 38.3^∗^	408.7 ± 9.6^∗^
AIHP (mg/plate)					
0.3125	32.3 ± 2.5	128.6 ± 4.7	17.3 ± 3.3	11.0 ± 1.4	180.0 ± 5.1
0.625	25.7 ± 5.7	131.6 ± 4.7	17.3 ± 3.3	10.3 ± 2.4	182.3 ± 4.5
1.25	29.3 ± 1.2	133.0 ± 3.3	16.0 ± 2.4	9.0 ± 1.4	184.7 ± 2.1
2.5	30.3 ± 1.2	132.0 ± 3.6	17.7 ± 2.6	10.7 ± 3.3	180.7 ± 7.5
5	32.7 ± 1.7	130.0 ± 2.9	21.0 ± 2.9	10.3 ± 2.6	181.6 ± 4.7

^a^Control group was added with dd H_2_O.

^b^Positive reagents without S‐9 mix reactions were 2.5 *μ*g/plate 4‐nitroquinoline‐N‐oxide for TA98, 5 *μ*g/plate sodium azide for TA100, 5 *μ*g/plate sodium azide for TA1535, 2.5 *μ*g/plate 4‐nitroquinoline‐N‐oxide for WP2 *uvr* A (pKM101), and 50 *μ*g/plate 9‐aminoacridine for TA1537; positive reagent with S‐9 mix was 5 *μ*g/plate 2‐aminoanthracene for all strains.

^c^Data was presented as mean ± SD (*n* = 3).

^∗^^∗^Significant difference of colonies more than two folds of negative control and treated groups at *p* < 0.05.

### 3.2. AIHP Did Not Induce Chromosomal Aberration in CHO‐K1 Cells

The cytotoxicity assay was strategically performed to determine optimal concentrations for the subsequent chromosomal aberration assay, ensuring the scientific rigor of our approach. Cell viability was not reduced at a concentration of 5 mg/mL. AIHP was comprehensively tested at 0.3125, 0.625, 1.25, 2.5, and 5 mg/mL. In this robust assessment, CHO‐K1 cells were exposed to AIHP across all concentrations, both with and without S9 metabolic activation. Impressively, chromosomal aberration frequencies in all AIHP‐treated groups remained statistically indistinguishable from the control group, providing evidence of a lack of genotoxicity. As anticipated, positive control treatments resulted in a marked and statistically significant rise in chromosomal aberration rates under both S9‐activated and non‐activated conditions (Table 2), validating the sensitivity and reliability of the assay. These findings strongly reinforce the exceptional genetic safety profile of AIHP.

**Table 2:** Percentages of chromosomal aberration test after incubation with AIHP in the cultured CHO‐K1 cells with or without S9 for 24 h.

Group	Total aberrations	Frequency of chromosomal aberration (%)^a^
Without S9 (‐S9)		
Control	11/300	3.7 ± 2.1^b^
Mitomycin C (2.5 *μ*g/mL)	66/300	22.0 ± 2.6^∗^
AIHP (mg/mL)		
0.3125	14/300	4.7 ± 3.0
0.625	9/300	3.0 ± 0.0
1.25	12/300	4.0 ± 1.0
2.5	8/300	2.7 ± 1.2
5	12/300	4.0 ± 2.6
With S9 (+S9)		
Control	7/300	2.7 ± 1.5
Cyclophosphamide (25 *μ*g/mL)	81/300	27.0 ± 1.7^∗^
AIHP (mg/mL)		
0.3125	10/300	3.3 ± 1.2
0.625	9/300	3.0 ± 1.0
1.25	7/300	2.3 ± 1.2
2.5	10/300	3.3 ± 3.2
5	8/300	2.7 ± 1.5

^a^Two slides were prepared and stained with diff Quik kit for 3 steps and a total number of 300 metaphases were counted for each dosage. All results were expressed in number of aberrations per plate.

^b^The number of cells with damage chromosomes was recorded from which the rate of mutation was calculated. Aberration rate (*%*) = (number of cells with damage chromosomes/100) × 100. Data are expressed as mean ± SD (*n* = 3).

^∗^^∗^Significant difference between the negative control and treated groups at *p* < 0.05.

### 3.3. AIHP Did Not Induce Erythrocyte Micronucleus in ICR Mice

Crucially, no treatment‐related adverse clinical signs or mortality were observed in any group following AIHP administration, underscoring its outstanding safety profile. As expected, positive control mice treated with cyclophosphamide (60 mg/kg, i.p.) displayed a dramatic and statistically significant increase in Mn‐RET frequencies—26.4 ± 4.0 at 48 h and 25.4 ± 5.7 at 72 h—compared with negative controls, thereby validating the sensitivity of the assay. In striking contrast, AIHP administration at all tested doses (low, medium, and high) resulted in no significant changes in micronucleus frequency relative to the control group (Table 3), providing evidence that AIHP does not induce genotoxic effects. These results strongly reinforce the genetic safety and tolerability of AIHP in vivo.

**Table 3:** Changes of reticulocytes with micronuclei in the peripheral blood of male mice after orally treatment with AIHP.

Group/Intervals	Dose (mg/kg)	RETs/1000RBCs (‰)	Mn‐RETs/1000RETs (‰)
48 h			
C^a^	0	26.8 ± 3.6	1.6 ± 1.3
PC	60	7.4 ± 4.0^∗^	26.4 ± 4.0^∗^
AIHP	2000	29.0 ± 5.3	1.0 ± 0.7
	4000	25.6 ± 5.3	1.2 ± 0.8
	8000	27.0 ± 7.0	0.8 ± 0.8
72 h			
C	0	27.2 ± 6.5	2.0 ± 0.7
PC	60	7.2 ± 1.9^∗^	25.4 ± 5.7^∗^
AIHP	2000	26.4 ± 6.8	2.0 ± 1.0
	4000	26.2 ± 4.4	2.8 ± 1.9
	8000	30.2 ± 5.8	1.8 ± 1.9

Abbreviations: C: control; Mn‐RETs: micronucleated reticulocytes; PC: positive control (Cyclophosphamide 60 mg/kg bw. ip); RBCs: erythrocytes; RETs: reticulocytes.

^a^Data are expressed as the mean ± SD (*n* = 5).

^∗^^∗^Significant difference in compared with the negative control and treated groups at *p* < 0.05.

### 3.4. Clinical Observations in the Repeated Dose 28‐Day Oral Toxicity Study in Rats: No Treatment‐Related Effects Observed

Remarkably, no toxicity‐related clinical signs or mortality were observed in any group throughout the entire 28‐day administration of AIHP, demonstrating its superior safety and tolerability. Furthermore, weekly body weight assessments consistently revealed no significant differences between treated and control groups in either sex, reinforcing the absence of adverse effects and supporting the excellent safety profile of AIHP during prolonged use (Figure 1).

Figure 1Body weight changes of rats in the 28‐day oral toxicity study of AIHP. Body weight changes are listed in male (a) and female (b) rats. The control group, the low dose group (2000 mg/kg body weight), the middle dose group (4000 mg/kg body weight) and the high dose group (8000 mg/kg body weight).

**Figure:**
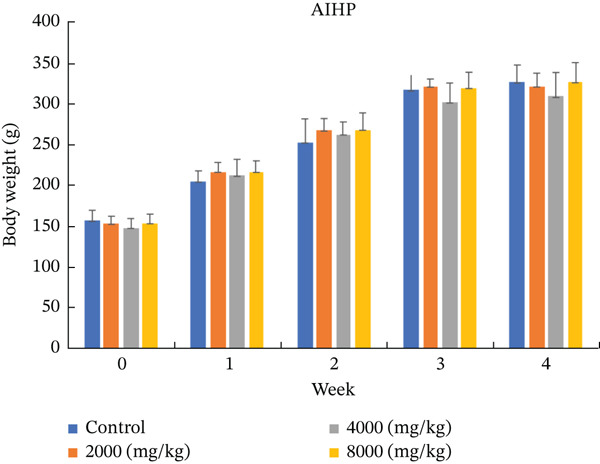
(a)

**Figure:**
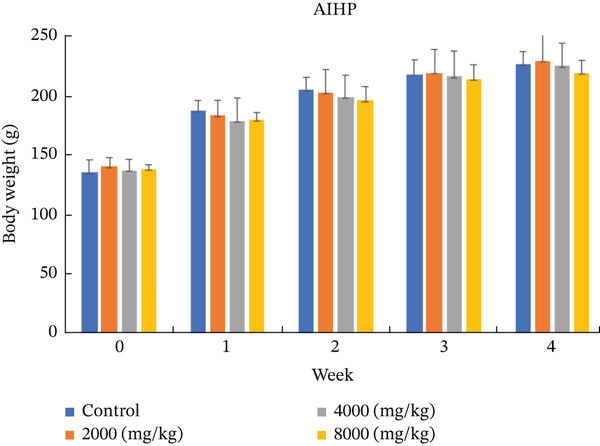
(b)

### 3.5. No Significant Changes Observed in Urinalysis

Urine samples were collected on Day 28 and rigorously analyzed for a comprehensive set of parameters, including color, specific gravity, bilirubin, urobilinogen, pH, protein, glucose, ketones, nitrites, occult blood, leukocytes, red blood cells, casts, and crystals (Table S1). Impressively, no significant or abnormal alterations were detected in any urinalysis parameter throughout the entire study across all AIHP treatment groups. Urinalysis parameters were evaluated qualitatively. No treatment‐related or abnormal findings were observed in any group, and all results were within the range of normal physiological variation. These consistently normal findings provide powerful support for the renal safety and physiological compatibility of AIHP, even with repeated administration.

### 3.6. No Significant Changes in Hematology and Serum Biochemistry Parameters

Hematological parameters (Table 4) and white blood cell differentials (Table 5) revealed no significant differences between AIHP‐treated groups and controls, underscoring the hematological safety of AIHP. While serum biochemistry analysis identified some statistically significant differences from control values (Table 6) including increases in amylase and sodium in male rats, elevated chloride in medium‐ and high‐dose males, and higher phosphate in high‐dose females all values remained well within established physiological reference ranges [22]. These minor fluctuations were not considered treatment‐related, further reinforcing the excellent systemic safety and physiological compatibility of AIHP, even at elevated doses. Collectively, these findings provide evidence for the absence of adverse hematological or biochemical effects associated with AIHP administration.

**Table 4:** Hematological parameter changes of rats in the 28‐day oral toxicity study of AIHP.

Sex/group	RBC^a^ (10^6^/*μ*L)	HGB (g/dL)	HCT (%)	MCV (fL)	MCH (pg)	MCHC (g/dL)	Platelets (10^3^/*μ*L)
Male							
Control	7.9 ± 0.8^b^	15.2 ± 1.2	46.8 ± 3.6	59.6 ± 2.5	19.4 ± 0.7	32.6 ± 0.6	1041.1 ± 226.3
AIHP							
2000 mg/kg	7.9 ± 1.5	15.9 ± 3.0	48.4 ± 9.2	61.0 ± 2.4	20.1 ± 0.7	32.9 ± 0.6	956.9 ± 385.9
4000 mg/kg	7.6 ± 0.4	14.8 ± 1.2	45.2 ± 3.3	59.6 ± 2.4	19.5 ± 0.9	32.8 ± 0.9	1018.6 ± 212.9
8000 mg/kg	8.3 ± 1.9	16.1 ± 3.7	49.4 ± 11.9	59.4 ± 1.5	19.4 ± 0.5	32.7 ± 0.5	946.3 ± 269.3
Female							
Control	7.8 ± 1.1	14.8 ± 2.1	44.9 ± 6.4	57.9 ± 1.5	19.1 ± 0.7	33.0 ± 0.7	953.3 ± 239.4
AIHP							
2000 mg/kg	7.5 ± 0.5	14.6 ± 0.8	43.8 ± 2.3	58.7 ± 1.7	19.6 ± 0.6	33.3 ± 0.6	933.1 ± 228.6
4000 mg/kg	7.5 ± 0.9	14.3 ± 1.6	43.4 ± 4.9	58.0 ± 1.8	19.0 ± 0.4	32.8 ± 0.6	929.5 ± 345.1
8000 mg/kg	7.5 ± 2.1	14.7 ± 3.9	43.9 ± 12.8	58.5 ± 2.1	19.8 ± 1.0	33.8 ± 2.1	834.2 ± 418.1

^a^HCT, hematocrit; HGB, hemoglobin; MCH, mean corpuscular hemoglobin; MCHC, mean corpuscular hemoglobin concentration; MCV, mean corpuscular volume; RBC, red blood cell.

^b^Data are expressed as the mean ± SD (*n* = 10).

^∗^^∗^Significant difference between the control and treated groups at *p* < 0.05.

**Table 5:** White blood differentiation changes of rats in the 28‐day oral toxicity study of AIHP.

Sex/group	WBC^a^	Differential leukocyte count (%)				
	(10^3^/*μ*L)	Lymphocyte	Neutrophil	Monocyte	Eosinophil	Basophil
Male						
Control	1.9 ± 0.7^b^	79.8 ± 7.5	17.3 ± 7.6	0.8 ± 0.3	2.2 ± 1.6	0.0 ± 0.0
AIHP						
2000 mg/kg	1.9 ± 1.2	77.4 ± 14.2	19.7 ± 13.2	0.8 ± 0.8	1.9 ± 1.4	0.1 ± 0.1
4000 mg/kg	2.0 ± 0.8	75.8 ± 9.9	21.0 ± 10.3	1.0 ± 0.6	2.2 ± 2.7	0.0 ± 0.0
8000 mg/kg	2.6 ± 1.4	83.7 ± 7.9	13.9 ± 6.3	0.8 ± 0.5	1.4 ± 1.8	0.0 ± 0.2
Female						
Control	0.9 ± 0.7	75.5 ± 7.1	18.9 ± 6.7	1.6 ± 1.6	3.9 ± 3.5	0.0 ± 0.1
AIHP						
2000 mg/kg	0.7 ± 0.4	77.4 ± 5.4	16.7 ± 3.9	1.8 ± 1.0	4.1 ± 3.2	0.0 ± 0.0
4000 mg/kg	1.1 ± 0.6	80.1 ± 5.6	16.0 ± 5.7	1.5 ± 1.1	2.4 ± 1.1	0.0 ± 0.2
8000 mg/kg	1.4 ± 1.2	78.5 ± 7.8	17.1 ± 7.4	1.4 ± 0.6	2.9 ± 1.9	0.1 ± 0.2

^a^WBC: white blood cell.

^b^Data are expressed as the mean ± SD (*n* = 10).

^∗^^∗^Significant difference between the control and treated groups at *p* < 0.05.

**Table 6:** Serum biochemistry changes of rats in the 28‐day oral toxicity study of AIHP.

Sex		Male			Female			
Group/Item	Control	AIHP			Control	AIHP		
		2000 mg/kg	4000 mg/kg	8000 mg/kg		2000 mg/kg	4000 mg/kg	8000 mg/kg
AST^a^	87.9 ± 14.9^b^	98.6 ± 38.6	94.6 ± 19.5	91.4 ± 12.5	87.7 ± 13.8	93.1 ± 18.1	91.0 ± 11.4	100.6 ± 18.1
ALT	28.1 ± 4.5	28.4 ± 5.3	32.0 ± 6.7	30.5 ± 6.3	24.7 ± 3.5	24.2 ± 4.5	24.5 ± 5.2	25.6 ± 5.7
BUN	16.4 ± 3.4	16.5 ± 2.9	17.7 ± 2.5	17.1 ± 2.4	19.6 ± 2.9	20.0 ± 2.7	21.2 ± 3.2	22.7 ± 2.5^∗^
Creatinine	0.2 ± 0.1	0.2 ± 0.0	0.2 ± 0.0	0.2 ± 0.0	0.3 ± 0.1	0.3 ± 0.1	0.3 ± 0.1	0.3 ± 0.0
CK	316.4 ± 165.7	376.9 ± 300.8	297.9 ± 73.5	290.9 ± 158.5	281.8 ± 131.4	361.8 ± 201.8	281.6 ± 95.2	385.1 ± 191.0
Cholesterol	49.0 ± 9.0	47.7 ± 8.3	53.4 ± 11.7	44.2 ± 9.3	53.0 ± 11.4	45.5 ± 9.3	52.3 ± 13.7	54.3 ± 13.6
Amylase	1877.8 ± 287.9	1601.0 ± 165.4^∗^	1514.5 ± 222.0^∗^	1548.2 ± 292.6^∗^	1226.6 ± 721.6	912.3 ± 190.7	1089.5 ± 170.5	992.8 ± 131.8
Glucose	178.8 ± 48.4	161.5 ± 18.9	161.9 ± 20.5	162.7 ± 18.5	134.4 ± 23.3	142.6 ± 17.3	150.5 ± 30.9	147.0 ± 18.2
LDH	432.4 ± 312.6	422.5 ± 185.5	430.1 ± 137.0	457.0 ± 331.0	444.1 ± 252.7	614.6 ± 455.1	417.2 ± 77.4	704.4 ± 457.9
GGT	4.6 ± 1.4	3.6 ± 1.4	5.1 ± 2.2	4.1 ± 1.6	4.0 ± 1.9	4.9 ± 1.1	3.8 ± 1.5	3.4 ± 2.2
UA	1.6 ± 0.2	1.7 ± 0.5	1.5 ± 0.3	1.5 ± 0.2	1.4 ± 0.2	1.4 ± 0.3	1.3 ± 0.4	1.4 ± 0.4
Albumin	3.9 ± 0.2	3.8 ± 0.2	3.9 ± 0.2	3.9 ± 0.2	4.1 ± 0.1	4.1 ± 0.3	4.1 ± 0.2	4.1 ± 0.1
HDL‐C	10.2 ± 3.2	9.5 ± 2.0	11.5 ± 2.2	9.3 ± 2.3	13.2 ± 3.1	11.6 ± 2.5	13.0 ± 2.9	13.9 ± 3.7
TG	25.6 ± 6.2	26.3 ± 8.1	31.5 ± 10.1	22.1 ± 3.9	25.7 ± 4.6	25.7 ± 4.3	26.7 ± 4.2	29.4 ± 10.1
TP	5.6 ± 0.3	5.6 ± 0.3	5.6 ± 0.3	5.7 ± 0.2	6.0 ± 0.2	5.9 ± 0.4	5.9 ± 0.2	5.9 ± 0.2
Ca^2+^	10.2 ± 0.8	9.8 ± 0.3	9.8 ± 0.3	9.9 ± 0.3	9.9 ± 0.2	9.8 ± 0.2	9.8 ± 0.2	9.9 ± 0.3
Cl^-^	102.8 ± 1.3	103.7 ± 1.3	104.4 ± 1.1^∗^	105.5 ± 1.2^∗^	104.7 ± 1.8	104.4 ± 1.3	105.4 ± 1.6	105.7 ± 1.8
K^+^	5.6 ± 0.6	5.5 ± 0.8	5.2 ± 0.4	5.0 ± 0.5^∗^	5.1 ± 0.6	5.1 ± 1.0	4.8 ± 0.4	5.3 ± 0.8
Mg^2+^	2.2 ± 0.2	2.3 ± 0.3	2.3 ± 0.2	2.3 ± 0.2	2.4 ± 0.2	2.4 ± 0.2	2.5 ± 0.3	2.5 ± 0.2
Na^+^	141.4 ± 2.0	143.5 ± 1.9	143.4 ± 1.0	143.6 ± 0.9	142.0 ± 2.1	142.9 ± 2.1	143.3 ± 1.7	142.6 ± 2.2
Phosphates	8.5 ± 1.1	9.7 ± 1.6	9.4 ± 0.9	9.5 ± 1.3	8.5 ± 0.9	8.7 ± 1.1	9.1 ± 1.2	9.4 ± 0.8^∗^

^a^Albumin (g/dL); ALT: alanine aminotransferase (U/L); amylase (U/L); AST: aspartate aminotransferase (U/L); BUN: blood urea nitrogen (mg/dL); Ca^2+^: calcium (mg/dL); chloride (mEq/dL); cholesterol (mg/dL); creatine (mg/dL); CK: creatine kinase (U/L); Cl^−^: glucose (mg/dL); GGT: gamma glutamyl‐transferase (U/L); HDL‐C: high density lipids‐cholesterol (mg/dL); LDH: lactate dehydrogenase (U/L); K^+^: potassium (mEq/dL); Mg^2+^: magnesium (mg/dL); Na^+^: sodium; phosphates (mEq/dL); phosphates (mg/dL); TB: total bilirubin; TG: triglycerides (mg/dL); TP: total protein (g/dL); UA: uric acid (mg/dL).

^b^Data are expressed as the mean ± SD (*n* = 10).

^∗^^∗^Significant difference between the control and treated groups at *p* < 0.05.

### 3.7. Gross Pathology, Organ Weights, and Histopathology Revealed No Treatment‐Related Effects

Gross necropsy demonstrated the exceptional safety of AIHP, as no lesions attributable to the test substance or gross abnormalities were observed in any vital organs across all groups. After sacrifice, all organs were expertly excised and weighed, revealing that apart from a statistically significant decrease in heart weight in the high‐dose group (Table 7) and in the relative heart weights of female rats in the low‐ and high‐dose groups (Table 8), all other organ weight parameters remained entirely normal. Importantly, these isolated differences were deemed incidental, likely reflecting normal individual variation rather than any effect of AIHP administration. Comprehensive histopathological analysis further strengthened the safety profile, showing no treatment‐related lesions associated with AIHP (Figures S1–S4). The few non‐specific findings observed in a small number of animals (incidence 1/10) were clearly unrelated to the test substance (Figure S5). Collectively, these gross, organ weight, and histopathological findings provide supportive evidence for the outstanding systemic and organ‐specific safety of AIHP.

**Table 7:** Absolute organ weight changes of rats in the 28‐day oral toxicity study of AIHP.

Sex/group	Brain (g)	Heart (g)	Thymus (g)	Liver (g)	Kidney (g)	Adrenal (g)	Spleen (g)	Testes/ovaries (g)
Male								
Control	2.0 ± 0.3^a^	1.1 ± 0.1	0.5 ± 0.1	9.2 ± 0.9	2.6 ± 0.2	0.05 ± 0.01	0.5 ± 0.1	3.0 ± 0.1
AIHP								
2000 mg/kg	2.0 ± 0.1	1.1 ± 0.1	0.4 ± 0.1	9.1 ± 0.7	2.6 ± 0.3	0.05 ± 0.01	0.5 ± 0.1	3.2 ± 0.2
4000 mg/kg	2.0 ± 0.1	1.1 ± 0.1	0.5 ± 0.1	8.9 ± 1.2	2.5 ± 0.3	0.06 ± 0.01	0.5 ± 0.1	3.1 ± 0.2
8000 mg/kg	2.0 ± 0.1	1.1 ± 0.1	0.4 ± 0.1	9.1 ± 0.5	2.5 ± 0.2	0.06 ± 0.01	0.5 ± 0.1	3.0 ± 0.2
Female								
Control	1.9 ± 0.1	0.8 ± 0.0	0.4 ± 0.1	6.1 ± 0.5	1.6 ± 0.2	0.06 ± 0.01	0.4 ± 0.0	0.08 ± 0.01
AIHP								
2000 mg/kg	1.9 ± 0.1	0.8 ± 0.1	0.4 ± 0.1	6.1 ± 0.7	1.7 ± 0.2	0.06 ± 0.01	0.4 ± 0.1	0.08 ± 0.02
4000 mg/kg	1.9 ± 0.1	0.8 ± 0.1	0.4 ± 0.1	6.2 ± 0.8	1.7 ± 0.2	0.10 ± 0.00	0.4 ± 0.1	0.10 ± 0.00
8000 mg/kg	1.9 ± 0.1	0.7 ± 0.1^∗^	0.4 ± 0.1	5.9 ± 0.7	1.6 ± 0.1	0.06 ± 0.01	0.4 ± 0.0	0.07 ± 0.02

^a^Data are expressed as the mean ± SD (*n* = 10).

^∗^^∗^Significant difference between the control and treated groups at *p* < 0.05.

**Table 8:** Relative organ weight changes of rats in the 28‐day oral toxicity study of AIHP.

Sex/group	Brain (%)	Heart (%)	Thymus (%)	Liver (%)	Kidney (%)	Adrenal (%)	Spleen (%)	Testes/ovaries (%)
Male								
Control	0.6 ± 0.1^a^	0.4 ± 0.1	0.2 ± 0.0	2.9 ± 0.4	0.8 ± 0.1	0.02 ± 0.00	0.2 ± 0.0	1.0 ± 0.1
AIHP								
2000 mg/kg	0.6 ± 0.0	0.3 ± 0.0	0.1 ± 0.0	2.8 ± 0.2	0.8 ± 0.1	0.02 ± 0.00	0.2 ± 0.0	1.0 ± 0.1
4000 mg/kg	0.6 ± 0.1	0.3 ± 0.0	0.1 ± 0.0	2.9 ± 0.2	0.8 ± 0.1	0.02 ± 0.00	0.2 ± 0.1	1.0 ± 0.1
8000 mg/kg	0.6 ± 0.1	0.4 ± 0.1	0.1 ± 0.0	2.9 ± 0.3	0.8 ± 0.1	0.02 ± 0.00	0.2 ± 0.0	1.0 ± 0.1
Female								
Control	0.9 ± 0.1	0.4 ± 0.0	0.2 ± 0.0	2.7 ± 0.2	0.7 ± 0.1	0.03 ± 0.00	0.2 ± 0.0	0.03 ± 0.01
AIHP								
2000 mg/kg	0.8 ± 0.1	0.3 ± 0.0^∗^	0.2 ± 0.0	2.6 ± 0.1	0.7 ± 0.0	0.03 ± 0.00	0.2 ± 0.0	0.03 ± 0.01
4000 mg/kg	0.9 ± 0.1	0.4 ± 0.0	0.2 ± 0.0	2.7 ± 0.2	0.8 ± 0.0	0.03 ± 0.00	0.2 ± 0.0	0.04 ± 0.01
8000 mg/kg	0.8 ± 0.1	0.3 ± 0.0^∗^	0.2 ± 0.1	2.6 ± 0.4	0.7 ± 0.1	0.03 ± 0.01	0.2 ± 0.0	0.03 ± 0.01

^a^Data are expressed as the mean ± SD (*n* = 10).

^∗^^∗^Significant difference between the control and treated groups at *p* < 0.05.

## 4. Discussion

Evidence from previous studies demonstrates that AIHP exerts a potent anti‐inflammatory effect by markedly downregulating the inflammatory markers TNF‐*α*, IL‐1*β*, and NF‐*κ*B in the gastric mucosa of ethanol‐exposed rats [5]. These molecular markers are widely recognized as critical instigators of inflammation and ulcer formation, and their substantial suppression by AIHP highlights its impressive therapeutic potential. Such targeted modulation of inflammatory responses underscores AIHP’s capacity to disrupt proinflammatory pathways central to gastric ulcer development. Additionally, a marked decrease in COX‐2 expression further affirms AIHP’s ability to limit the production of harmful inflammatory mediators within gastric tissue [23]. These results are strongly supported by the established anti‐inflammatory effects of leading plant‐based compounds like curcumin [24], renowned for their inhibition of TNF‐*α*, IL‐1*β*, and NF‐*κ*B in diverse inflammatory diseases. The pathophysiological relevance is clear: excessive gastric acid secretion or reflux can devastate the gastric mucosa and activate NF‐*κ*B [25], which then amplifies the secretion of proinflammatory mediators and drives conditions such as gastritis and gastric ulcer [26]. As a pivotal regulator of both the inflammatory response and mucosal self‐repair [25], NF‐*κ*B represents a crucial therapeutic target. By inhibiting NF‐*κ*B activation, AIHP offers a highly promising strategy to alleviate gastric mucosal injury and accelerate healing in the context of gastric hyperacidity [27]. These mechanistic insights position AIHP at the forefront of innovative natural interventions for gastric inflammation and ulcer prevention.

AIHP demonstrated efficacy in inhibiting the overexpression of key inflammatory markers including TNF‐*α*, IL‐1*β*, COX‐2, and NF‐*κ*B in a pyloric ligation‐induced ulcer model in rats. These inflammatory mediators are well‐established drivers of gastric ulcer pathogenesis and its associated inflammatory cascade [28]. By dramatically suppressing these core markers, AIHP delivers robust protection at the molecular level, decisively interrupting the pathways that fuel inflammation and ulcer formation. The targeted inhibition of the NF‐*κ*B pathway highlights AIHP’s ability to disrupt critical mechanisms at the root of gastric inflammation and injury [29, 30]. Furthermore, the pronounced downregulation of TNF‐*α* and IL‐1*β* expression underscores the exceptional anti‐inflammatory power of AIHP. These results are consistent with those seen for leading natural therapeutics such as curcumin and licorice [31, 32], which are celebrated for their potent suppression of inflammatory markers in various gastric disorders. Collectively, these findings position AIHP as a highly promising and innovative solution for the prevention and management of gastric ulcers through its superior molecular anti‐inflammatory action.

Previous studies on *A. indica* have mainly focused on pharmacological efficacy, such as gastroprotective and anti‐inflammatory effects, while safety assessments were often limited to acute exposure or indirect assumptions based on traditional use. In contrast, the present study offers the first comprehensive toxicological evaluation of an *A. indica*‐containing formulation using a full OECD‐compliant genotoxicity battery combined with a repeated‐dose 28‐day oral toxicity study. This integrated approach enables definition of a clear NOAEL and provides regulatory‐relevant safety data that extend beyond efficacy‐oriented investigations. Our comprehensive evaluation provides evidence that AIHP is exceptionally safe, with no genotoxic effects detected in the Ames test, chromosomal aberration assay, or micronucleus assay in mice. Although some serum biochemical parameters showed statistically significant differences compared with controls, all values remained within established historical control ranges for SD rats and did not exhibit a dose–response relationship. Therefore, these findings were considered incidental rather than toxicologically relevant [22]. Importantly, these minimal alterations showed no dose‐dependency and were unaccompanied by any clinical abnormalities or histopathological signs of toxicity. Taken together, these robust findings demonstrate that AIHP poses no genotoxic or subacute toxicological risks at tested doses. The NOAEL of AIHP was confirmed at an impressive 8000 mg/kg in SD rats, strongly supporting its outstanding safety and suitability as a novel health food ingredient. Similar safety profiles have been reported for other Lamiaceae plants used as functional food ingredients, such as *Ocimum basilicum*, *Salvia officinalis*, and *Rosmarinus officinalis*, all of which are widely consumed and generally considered to have low systemic toxicity in animal studies. Acute and subchronic oral administration of *Ocimum basilicum* hydroalcoholic extract in rats caused no mortality or major clinical abnormalities [33]. Additionally, Lamiaceae species are rich in phenolic compounds and other antioxidant phytochemicals that have been associated with free‐radical scavenging and potential mitigation of oxidative or inflammatory stress [34]. The dose levels selected for the 28‐day oral toxicity study (2000, 4000, and 8000 mg/kg/day) were intentionally chosen to establish a conservative safety margin for AIHP as a health food ingredient, rather than as a pharmacologically active drug. As a food‐grade botanical powder formulation intended for dietary use, higher dose levels are commonly used in subacute toxicity testing to identify a clear no‐observed‐adverse‐effect level (NOAEL) and potential target organs under exaggerated exposure conditions.

The highest dose of 8000 mg/kg/day was selected as a practical limit dose, based on formulation feasibility and repeated‐dose tolerability during oral gavage, with no evidence of treatment‐related gastrointestinal intolerance or dosing volume constraints. Such high‐dose testing is considered appropriate for food ingredients and botanical products with anticipated low systemic toxicity, as it enables a robust assessment of safety margins relevant to human consumption. Using body surface area–based interspecies conversion, the NOAEL of 8000 mg/kg/day in rats corresponds to a human equivalent dose (HED) of approximately 1300 mg/kg/day. This conservative estimate greatly exceeds the anticipated daily intake of AIHP as a health food ingredient, indicating a wide margin of exposure. Collectively, these findings support the conclusion that AIHP does not pose systemic toxicity concerns under the intended conditions of human dietary use.

In conclusion, this comprehensive body of evidence demonstrates that AIHP is exceptionally safe, exhibiting no genotoxic or subacute toxicological risks under the tested conditions. The NOAEL for AIHP was confirmed at an impressive 8000 mg/kg in SD rats, supporting its promise and suitability as a novel, safe health food ingredient.

## Author Contributions

The conception and design of the study by Yng‐Tay Chen. Material preparation and data analysis were performed by Yng‐Tay Chen and Yu‐Ru Chen. Fuu‐Jen Tsai contributed to data interpretation, supervision, and manuscript review. Ter‐Hsin Chen provided project oversight, critical revisions, and final approval of the manuscript.

## Funding

No funding was received for this manuscript.

## Disclosure

All authors have read and approved the final version of the manuscript.

## Ethics Statement

The animal experiments were fulfilled according to the Guide for the Care and Use of Laboratory Animals. All protocols were approved by the Institutional Animal Care and Use Committee (IACUC) of the National Chung Hsing University in 2024, Taiwan (IACUC 113‐001).

## Conflicts of Interest

The authors declare no conflicts of interest.

## Supporting information


**Supporting Information** Additional supporting information can be found online in the Supporting Information section. Figure S1 Urinalysis changes of rats treated with AIHP in the 28‐day oral toxicity study. Figure S1. Histopathological changes of control rats in the 28‐day oral toxicity study of AIHP. Figure S2. Histopathological changes of the low dose group in the 28‐day oral toxicity study of AIHP. Figure S3. Histopathological changes of the middle dose group in the 28‐day oral toxicity study of AIHP. Figure S4. Histopathological changes of the high dose group in the 28‐day oral toxicity study of AIHP. Figure S5. Nonspecific histopathological findings of rats in the 28‐day oral toxicity study of AIHP.

## Data Availability

The datasets generated and analyzed during the current study are available from the corresponding author upon reasonable request.
