# Supplementary material for: Comprehensive Genotoxicity and 28‐Day Oral Toxicity Evaluation Reveal Safety of a Standardized Anisomeles indica‐Containing Powder
Source: Biomed Res Int. 2026 Mar 13;2026:9966654. doi: 10.1155/bmri/9966654 (PMC13140803; doi:10.1155/bmri/9966654)
Supplement: Supplementary file 1 — Supporting Information Additional supporting information can be found online in the Supporting Information section. Figure S1 Urinalysis changes of rats treated with AIHP in the 28‐day oral toxicity study. Figure S1. Histopathological changes of control rats in the 28‐day oral toxicity study of AIHP. Figure S2. Histopathological changes of the low dose group in the 28‐day oral toxicity study of AIHP. Figure S3. Histopathological changes of the middle dose group in the 28‐day oral toxicity study of AIHP. Figure S4. Histopathological changes of the high dose group in the 28‐day oral toxicity study of AIHP. Figure S5. Nonspecific histopathological findings of rats in the 28‐day oral toxicity study of AIHP. [file BMRI-2026-9966654-s001.pdf]

Table S1. Urinalysis changes of rats treated with AIHP in the 28-day oral toxicity study

Sex/Group	pH	Specific gravity				Glucose[1]			Protein					
		1.015	1.020	1.025	>1.030	-	Trace	+	-	Trace	+	++	+++	++++
**Male**														
Control	7.1 ± 0.2[2]	1[3]	2	1	5	10	0	0	4	0	5	1	0	0
AIHP														
2000 mg/kg	7.3 ± 0.3	7	1	2	0	10	0	0	8	0	2	0	0	0
4000 mg/kg	7.3 ± 0.6	3	2	1	4	10	0	0	4	2	1	3	0	0
8000 mg/kg	7.3 ± 0.3	5	5	0	0	10	0	0	8	1	1	0	0	0
**Female**														
Control	7.0 ± 0.3	2	1	2	5	10	0	0	8	0	2	0	0	0
AIHP														
2000 mg/kg	7.1 ± 0.2	1	4	3	2	10	0	0	9	0	1	0	0	0
4000 mg/kg	7.0 ± 0.3	3	1	3	3	10	0	0	10	0	0	0	0	0
8000 mg/kg	7.0 ± 0.2	6	4	1	0	10	0	0	10	0	0	0	0	0

AIHP: *A. indica* HP813 Powder.

[1] Grades for each item: Glucose (mg/dL): negative (0); trace (50); + (100); ++ (200); +++ (500); ++++ (1000); protein (mg/dL): negative (0); trace (15); + (30); ++ (100); +++ (300); ++++ (1000).

[2] Data are expressed as the mean ± SD (n = 10)

[3] Number of rats were observed.

Table S1. Urinalysis changes of rats treated with AIHP in the 28-day oral toxicity study (cont.)

Sex/Group	Appearance[1]				Urobilinogen	Bilirubin		Nitrite		
	P	Y	DY	B	(mg/dL)	-	+	-	+	++
**Male**										
Control	0[2]	8	2	0	0.4 ± 0.4[3]	10	0	3	7	0
AIHP										
2000 mg/kg	0	10	0	0	0.2 ± 0.0	10	0	3	7	0
4000 mg/kg	0	8	2	0	0.3 ± 0.3	10	1	4	6	0
8000 mg/kg	0	10	0	0	0.2 ± 0.0	10	0	2	8	0
**Female**										
Control	0	8	2	0	0.3 ± 0.3	9	1	2	8	0
AIHP										
2000 mg/kg	0	9	1	0	0.2 ± 0.0	10	0	1	9	0
4000 mg/kg	0	10	0	0	0.2 ± 0.0	110	0	3	7	0
8000 mg/kg	0	10	0	0	0.2 ± 0.0	10	0	4	6	0

AIHP: *A. indica* HP813.

[1] Grades for each items: appearance: P (pale), Y (yellow), DY (dark yellow), B (brown); bilirubin (mg/dL): negative (0); + (0.5), ++ (2), +++ (6), ++++ (over); nitrite: - (normal); + (positive).

[2] Number of rats were observed.

[3] Data are expressed as the mean ± SD (n = 10).

[*] Significant difference between the control and treated groups at $p < 0.05$

Table S1. Urinalysis changes of rats treated with AIHP in the 28-day oral toxicity study (cont.)

Sex/Group	Oc. Blood[1]			Leukocyte					Ketone				
	-	trace	+	-	trace	+	++	+++	-	trace	+	++	+++
**Male**													
Control	10[2]	0	0	5	5	0	0	0	1	5	4	0	0
AIHP													
2000 mg/kg	10	0	0	10	0	0	0	0	5	5	0	0	0
4000 mg/kg	10	0	0	7	3	0	0	0	2	5	3	0	0
8000 mg/kg	10	0	0	8	2	0	0	0	5	5	0	0	0
**Female**													
Control	10	0	0	10	0	0	0	0	6	4	0	0	0
AIHP													
2000 mg/kg	10	0	0	10	0	0	0	0	7	3	0	0	0
4000 mg/kg	10	0	0	10	0	0	0	0	10	0	0	0	0
8000 mg/kg	10	0	0	10	0	0	0	0	10	0	0	0	0

AIHP: *A. indica* HP813 Powder.

[1] Grades for each items: Occult blood (oc. blood) (ery/μL): - (normal), + (20), ++(60), +++(300); leukocyte (leu/μL): - (0); trace(<25), + (25); ++ (75); +++ (250), ++++ (500); ketones (mg/dL): negative (0); trace (<15); + (15); ++ (40); +++ (80); ++++ (150).

[2] Number of rats were observed.

Table S1. Urinary changes of rats treated with AIHP in the 28-day oral toxicity study (cont.)

Sex/Group	Urinary segments[1]					
	RBC (hpf)			WBC (hpf)		
	0-2	3-5	6-15	0-2	3-5	6-15
**Male**						
Control	10[2]	0	0	10	0	0
AIHP						
2000 mg/kg	10	0	0	10	0	0
4000 mg/kg	10	0	0	10	0	0
8000 mg/kg	10	0	0	10	0	0
**Female**						
Control	10	0	0	10	0	0
AIHP						
2000 mg/kg	10	0	0	10	0	0
4000 mg/kg	10	0	0	10	0	0
8000 mg/kg	10	0	0	9	1	0

AIHP: *A. indica* HP813 Powder.

[1]Urinary sediments: RBC: red blood cells; WBC: white blood cells; hpf: high power field, 400x; 0-2 cell, 3-5 cells, 6-15, 6-50 cells, over 50 cells.

[2] Number of rats were observed.

Table S1. Urinary changes of rats treated with AIHP in the 28-day oral toxicity study (cont.)

Sex/Group	Cast[1] (lpf)		Crystal (lpf)			Bacteria(hpf)			
	-	+	None found	Triple phosphate	Calcium oxalate	-	+	++	+++
**Male**									
Control	10	0	0	10	0	0	5	2	3
AIHP									
2000 mg/kg	10	0	0	10	0	0	7	3	0
4000 mg/kg	10	0	1	7	2	0	4	4	2
8000 mg/kg	10	0	0	10	0	0	7	3	0
**Female**									
Control	10	0	1	8	1	0	5	4	1
AIHP									
2000 mg/kg	10	0	1	9	0	1	1	7	1
4000 mg/kg	10	0	0	9	1	0	5	5	0
8000 mg/kg	10	0	1	8	1	0	4	4	2

AIHP: *A. indica* HP813 Powder.

[1]Urinary sediments: Cast, crystal and parasite: - (none); + (present); lpf: low power filed, 100x; hpf: high power field, 400x.

[2] Number of rats were observed.

Histopathological examination

Organs collected from rats in the control, low-, medium-, and high-dose AIHP groups underwent meticulous histopathological evaluation to ensure a comprehensive assessment of safety. The full spectrum of vital organs—including adrenal glands, brain, heart, kidneys, liver, lungs, spleen, thymus, testes (male rats), and ovaries (female rats)—was rigorously examined. Each tissue was expertly processed: trimmed, embedded in paraffin, and sectioned at a precise thickness of 3 μm using a state-of-the-art rotary microtome (Leica RM 2245, Nussloch, Germany). Sections were stained with hematoxylin and eosin (H&E) and scrutinized under high-resolution light microscopy to identify any histopathological changes. Lesion severity was assessed according to the grading system proposed by Shackelford et al. (2002), which classifies lesions into five grades: grade 1 (<1%, minimal), grade 2 (1-25%, slight), grade 3 (26-50%, moderate), grade 4 (51-75%, moderately severe), and grade 5 (76-100%, severe). Notably, the histopathological evaluation revealed only isolated, minimal findings across all groups. Multifocal pelvic mononuclear cell infiltration was observed in just one male rat from the low-dose group (1/10), with the lesion classified as minimal (Figure S5F) and showing no association with AIHP administration. Similarly, multifocal tubular regeneration in the kidney of the same animal (Figure S5G) and minimal multifocal hemorrhage in the thymus of one female rat from the high-dose group (1/10; Figure S5H) were detected, both clearly unrelated to AIHP treatment. The rarity, minimal severity, and lack of dose-dependent occurrence powerfully reinforce the conclusion that AIHP administration does not produce treatment-related adverse histopathological effects. These results provide compelling evidence of the outstanding organ-specific safety of AIHP, even with repeated administration at high doses.

Reference:

Shackelford C, Long G, Wolf J, Okerberg C, Herbert R. Qualitative and quantitative analysis of nonneoplastic lesions in toxicology studies. *Toxicol Pathol.* 2002;30:93-96.

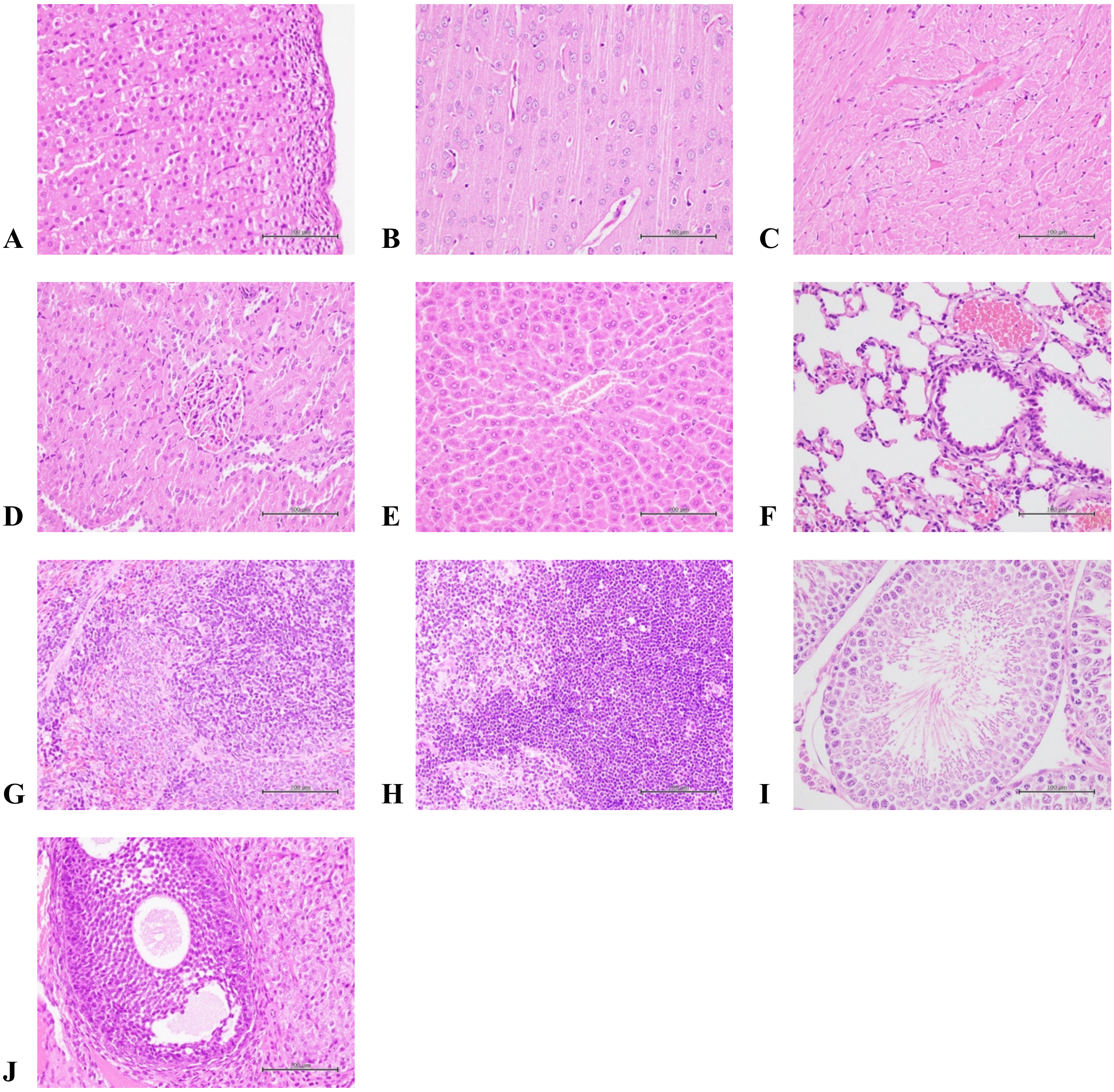

Figure S1. Histopathological changes of control rats in the 28-day oral toxicity study of AIHP. Adrenal glands (A), brain (B), heart (C), kidney (D), liver (E), lung (F), spleen (G), thymus (H), testis (I) (animal No.: 1001) and ovary (J) (animal No.: 1101) showed no histopathological changes in the control group (H&E stain, 400x).

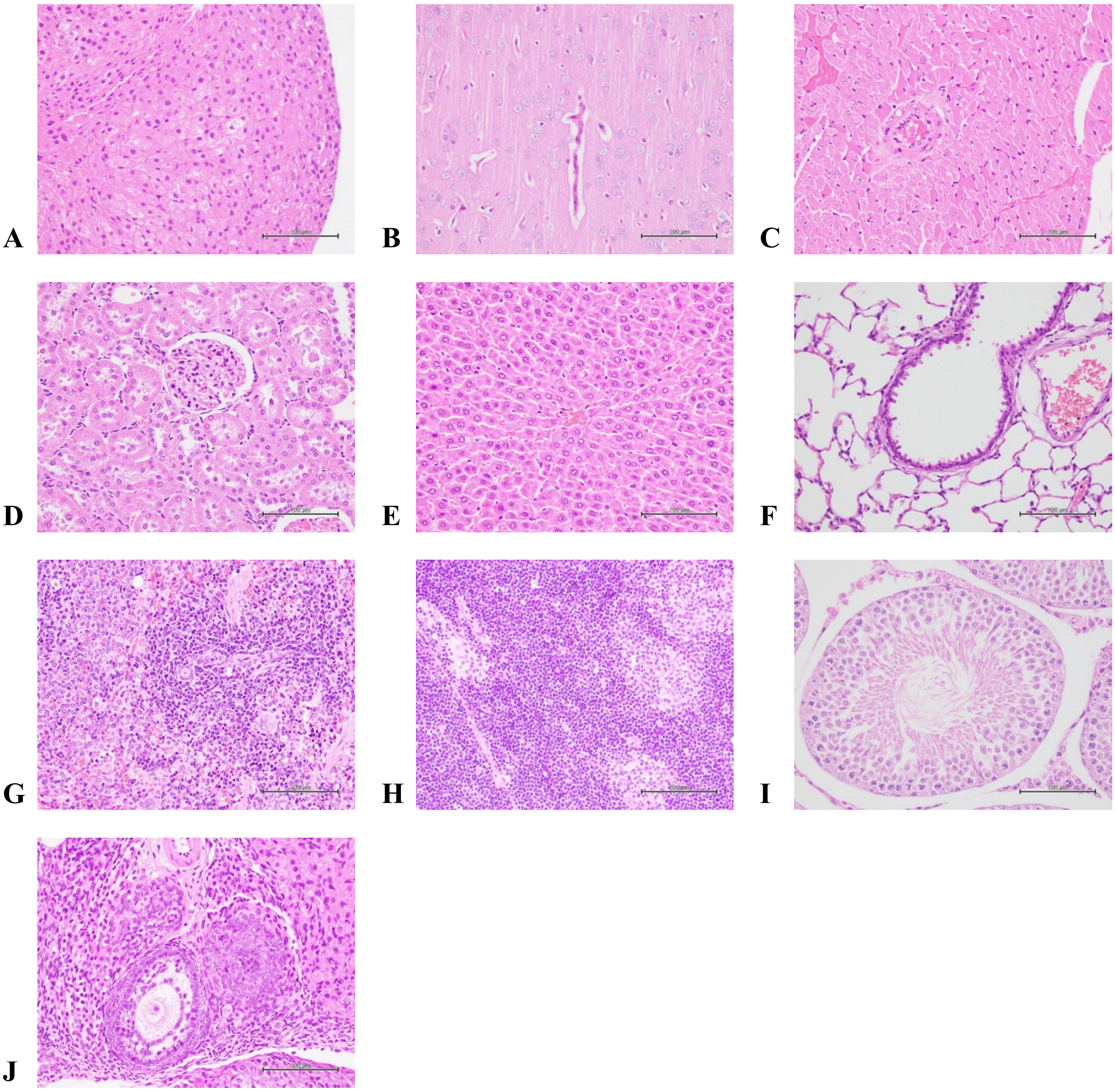

Figure S2. Histopathological changes of the low dose group in the 28-day oral toxicity study of AIHP. Adrenal glands (A), brain (B), heart (C), kidney (D), liver (E), lung (F), spleen (G), thymus (H), testis (I) (animal No.: 2001) and ovary (J) (animal No.: 2101) showed no histopathological changes in the low dose group (H&E stain, 400x).

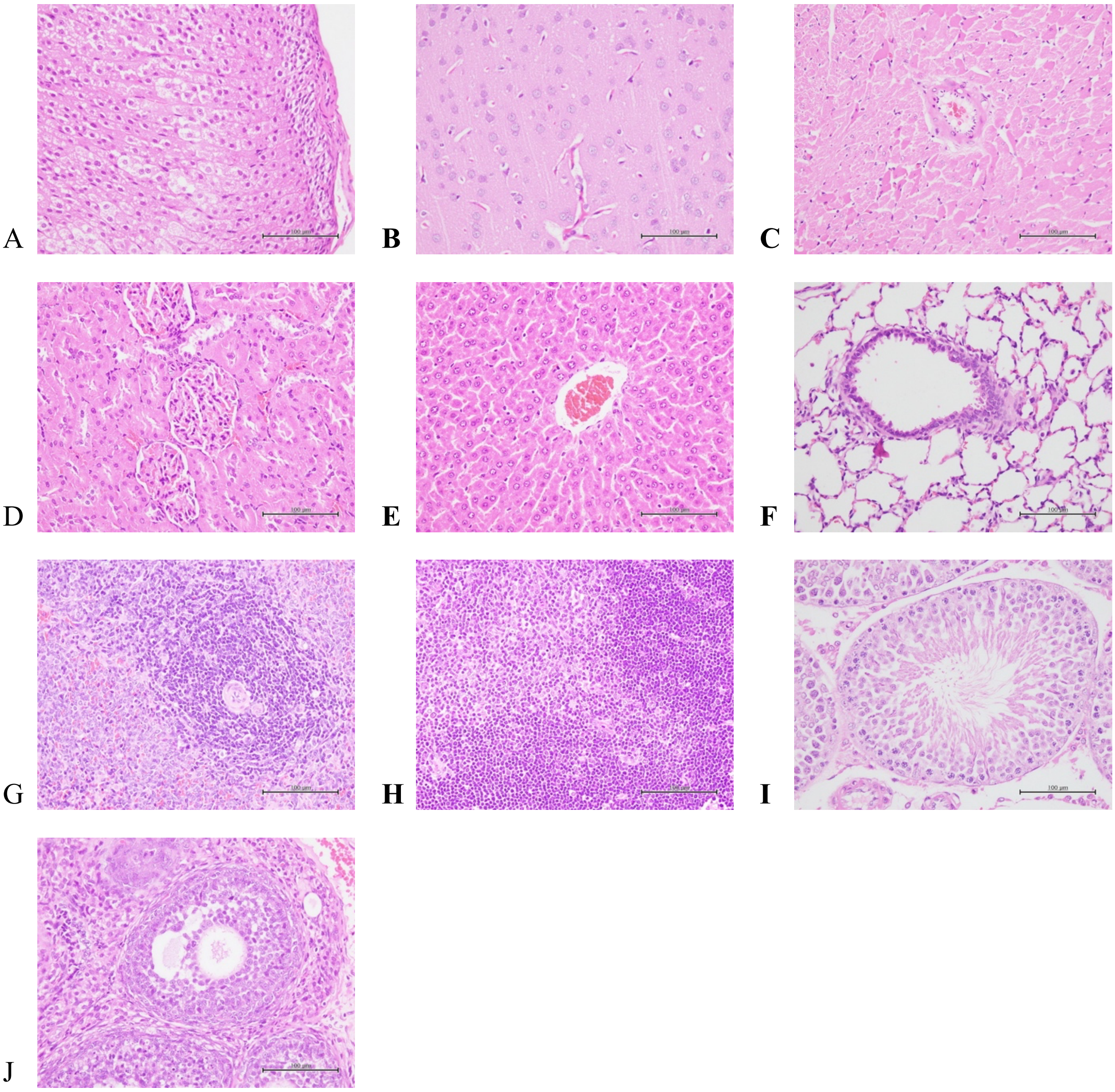

Figure S3. Histopathological changes of the middle dose group in the 28-day oral toxicity study of AIHP. Adrenal glands (A), brain (B), heart (C), kidney (D), liver (E), lung (F), spleen (G), thymus (H), testis (I) (animal No.: 3001) and ovary (J) (animal No.: 3101) showed no histopathological changes in the middle dose group (H&E stain, 400x).

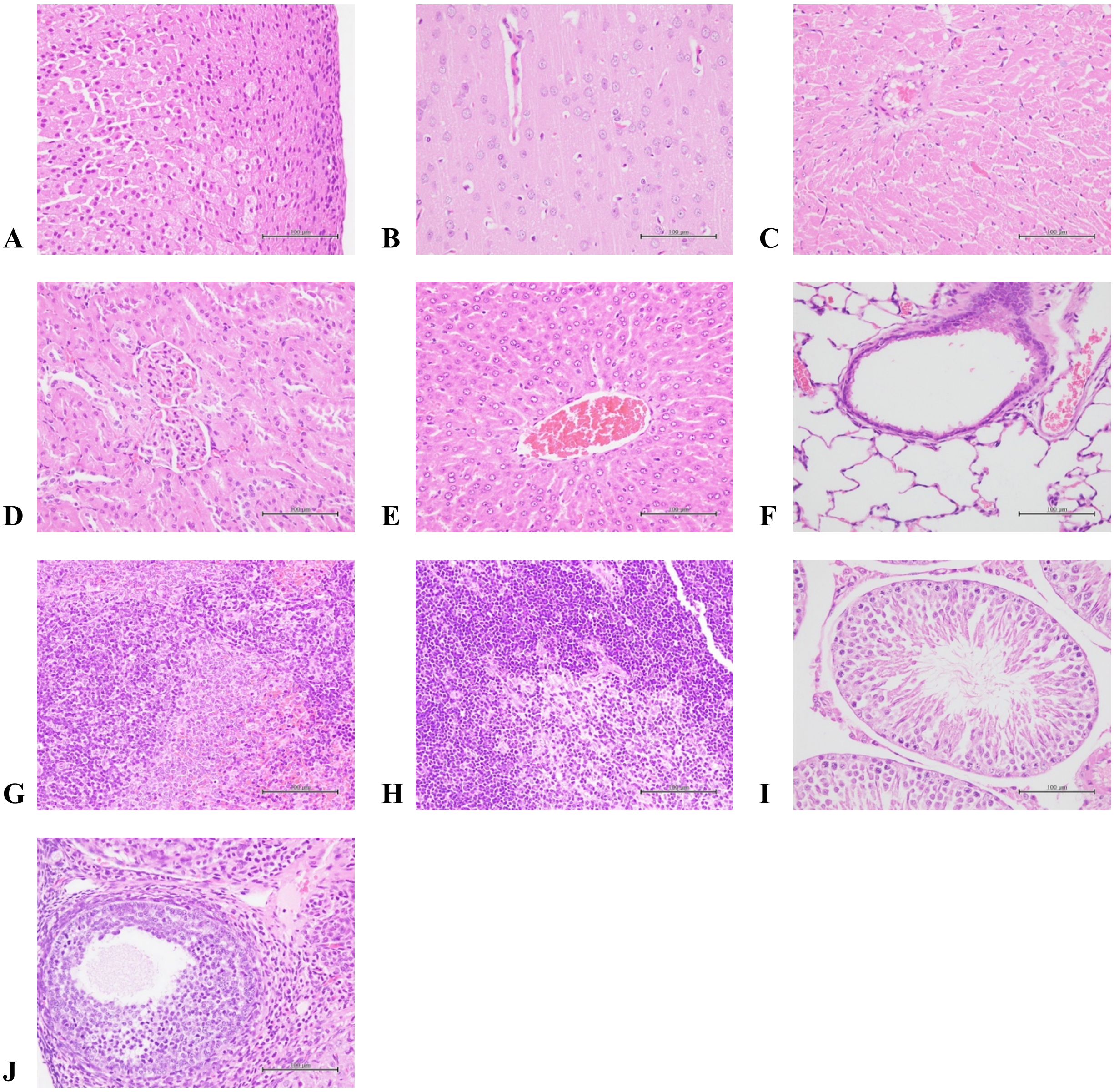

Figure S4. Histopathological changes of the high dose group in the 28-day oral toxicity study of AIHP. Adrenal glands (A), brain (B), heart (C), kidney (D), liver (E), lung (F), spleen (G), thymus (H), testis (I) (animal No.: 4001) and ovary (J) (animal No.: 4101) showed no histopathological changes in the high dose group (H&E stain, 400x).

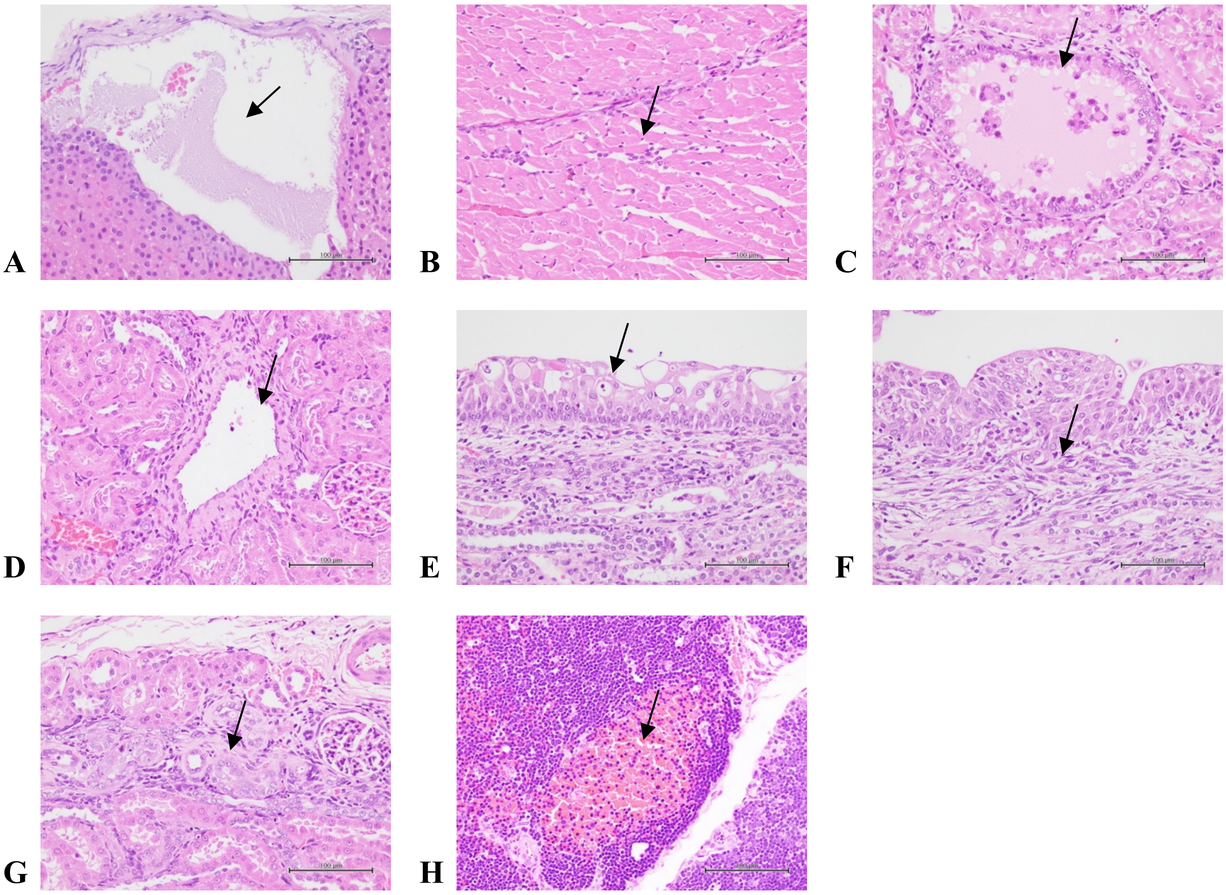

Figure S5. Non-specific histopathological findings of rats in the 28-day oral toxicity study of AIHP. Focal cyst in the adrenal glands (A. animal No.: 2009); focal mononuclear cell infiltration in the heart (B. animal No.: 1004); focal atypical tubular hyperplasia (C. animal No.: 1105), cyst (D. animal No.: 2005), epithelial hyperplasia (E), mononuclear cell infiltration (F) and tubular regeneration (G. animal No.: 3007) in the kidneys; multifocal hemorrhage in the thymus (H. animal No.: 4109) were found in rats (H&E stain, 400x). Arrow.
